# Trauma team leaders’ non-verbal communication: video registration during trauma team training

**DOI:** 10.1186/s13049-016-0230-7

**Published:** 2016-03-25

**Authors:** Maria Härgestam, Magnus Hultin, Christine Brulin, Maritha Jacobsson

**Affiliations:** Department of Nursing, Umeå University, S-90187 Umeå, Sweden; Department of Surgical and Perioperative Sciences, Umeå University, S-90185 Umeå, Sweden; Department of Social Work, Umeå University, S-90187 Umeå, Sweden

**Keywords:** Communication, Coordination, Leadership, Non-verbal communication, Time, Trauma team, Trauma team training

## Abstract

**Background:**

There is widespread consensus on the importance of safe and secure communication in healthcare, especially in trauma care where time is a limiting factor. Although non-verbal communication has an impact on communication between individuals, there is only limited knowledge of how trauma team leaders communicate. The purpose of this study was to investigate how trauma team members are positioned in the emergency room, and how leaders communicate in terms of gaze direction, vocal nuances, and gestures during trauma team training.

**Methods:**

Eighteen trauma teams were audio and video recorded during trauma team training in the emergency department of a hospital in northern Sweden. Quantitative content analysis was used to categorize the team members’ positions and the leaders’ non-verbal communication: gaze direction, vocal nuances, and gestures. The quantitative data were interpreted in relation to the specific context. Time sequences of the leaders’ gaze direction, speech time, and gestures were identified separately and registered as time (seconds) and proportions (%) of the total training time.

**Results:**

The team leaders who gained control over the most important area in the emergency room, the “inner circle”, positioned themselves as heads over the team, using gaze direction, gestures, vocal nuances, and verbal commands that solidified their verbal message. Changes in position required both attention and collaboration. Leaders who spoke in a hesitant voice, or were silent, expressed ambiguity in their non-verbal communication: and other team members took over the leader’s tasks.

**Discussion:**

In teams where the leader had control over the inner circle, the members seemed to have an awareness of each other’s roles and tasks, knowing when in time and where in space these tasks needed to be executed. Deviations in the leaders’ communication increased the ambiguity in the communication, which had consequences for the teamwork. Communication cannot be taken for granted; it needs to be practiced regularly just as technical skills need to be trained. Simulation training provides healthcare professionals the opportunity to put both verbal and non-verbal communication in focus, in order to improve patient safety.

**Conclusions:**

Non-verbal communication plays a decisive role in the interaction between the trauma team members, and so both verbal and non-verbal communication should be in focus in trauma team training. This is even more important for inexperienced leaders, since vague non-verbal communication reinforces ambiguity and can lead to errors.

## Background

There is a great awareness that effective verbal communication among team members in emergency situations plays an important role in the team’s performance [1–4]. However, non-verbal communication must also be taken into consideration since it represents more than 65 % of the communication [5]. Leadership has been described as another key factor in the trauma team’s performance [6–8]. The designated team leader is responsible for effective communication in order to supervise and coordinate the team members’ activities [9, 10].

Verbal and non-verbal communication have traditionally been studied separately as two independent factors to be taken into consideration in the interaction [11]. Most studies on trauma teamwork have focused on verbal communication, which can be interpreted as suggesting that non-verbal communication is of less importance [13–15]. Previous research on non-verbal communication in health care has primarily focused on doctor-patient interaction [16–18], while to our knowledge studies of interdisciplinary teams have focused mostly on anaesthesia teams [19–21] and surgical teams [22, 23]. In this article, our point of departure is that it is essential to explore verbal as well as non-verbal communication since they are both significant parts of communication [5], and both play an important role in social interaction [24].

Jones and LeBaron [11] have pointed out that, according to Kendon [12], it makes no sense to speak of verbal and non-verbal communication: there is only communication. When individuals communicate, they integrate their bodies in the interaction, for instance with postures, gaze direction, and gestures. Studies have shown that individuals’ positions in the room, gaze direction, vocal nuances, and gestures can provide important clues for understanding their degree of attention and involvement in the interaction [18, 22, 23]. A chosen posture can express an invitation to interact or mediate disinterest, which can affect how the interaction is experienced [18, 25, 26]. Earlier studies of interdisciplinary teams have shown that collaboration does not necessarily require verbal requests in order to get the work done [19, 21–23]. Delicate bodily shifts indicated a change in urgency of action and need for assistance in the collaboration in anaesthetic [21] and surgical teams [22, 23]. Directed by the tactile instructions, the team members shifted their positions, indicating awareness of the situation, and acted to help. The participants in the teams coordinated and synchronized their body movements based on their experience and knowledge of each other’s tasks, which became clear when inexperienced members participated [21]. Both verbal and non-verbal expressions are important for this coordination in interdisciplinary surgical and anaesthetic teamwork [27–29]. Body position together with gaze direction can enhance or weaken the communication [18]. The most important non-verbal communication is that mediated through the face, especially gaze direction [30]. Eye contact signals an invitation to join the conversation [25], and a turning-away of the gaze signals that attention is directed to another target [18, 31]. The voice is of importance since the speaker’s credibility plays a crucial role in convincing the audience [24]. Scherer points out the relationship between the features of the voice and their social impact [32]. More fluent speech without stuttering and a loud and clear voice will improve the impression of the speaker. The concept of vocal nuances such as speech time, intonation, vocal quality, and silence (cf. [24, 32]) will be used in this article. The focus is more on *how* something is said rather than *what* is said. Behaviours and voice changes during procedures are recognized and understood by members of the surgical team as indicating complications and urgency. Changes in tone, lifted eyebrows, and stretching postures send signals to other team members about important changes in the patient’s status [22, 23]. To facilitate (or hinder) the understanding of the spoken message, gestures can be directly tied to words in order to illustrate, emphasize, and point things out. Hands are seen as important carriers of information [33]. Gestures can be divided into speech-independent gestures and conversational gestures [24].

In this article, the focus is on team leaders’ communication, and the analysis is performed in relation to both verbal and non-verbal communication. As far as we have found, there is only limited knowledge of how non-verbal communication occurs in trauma teams. Thus, the aim of this study was to investigate how trauma team members are positioned in the emergency room, and how leaders communicate in terms of gaze direction, vocal nuances, and gestures during trauma team training.

## Methods

### Participants

The participants in the trauma teams consisted of personnel involved in regular trauma team training. A total of 108 participants were included (physicians *n* = 36, nurses *n* = 36, enrolled nurses *n* = 36), in 18 teams each consisting of six participants: one surgeon/emergency physician, one anaesthesiologist, one registered nurse anaesthetist, one registered nurse from the emergency department, one enrolled nurse from the emergency department, and one enrolled nurse from the operating theatre. A total of 65 (60 %) of the participants had experience of a previous trauma course 72 (67 %) had previous experience of trauma team training with video-facilitated debriefing, and 100 (93 %) of the participants had previous experience of trauma. The surgeon/emergency physician was the designated leader of the trauma team. The leaders’ median age was 40 years with an interquartile range (IQR) of 30–56, and their median time in profession was 4 years with an IQR of 2–26. Four of the leaders (22 %) were senior physicians, five of them (28 %) were women, and three (17 %) had a non-Scandinavian background.

Before attending the team training, the participants were asked to view a five-minute introductory video about teamwork in emergency settings, with the focus on collaboration and communication according to Crisis Resource Management (CRM) principles. The teams were included in the study after written consent was received from all participants. They were assured that they could leave the study whenever they wished to, and that the recorded material would be handled confidentiality. The study was approved by the Regional Ethical Review Board in Umeå (9 June 2009, ref: 09–106 M).

### Research setting

The trauma team training took place in the emergency room at the emergency department in a hospital in northern Sweden. The hospital is a level one trauma centre, and responsible for highly specialized care for the 877,000 inhabitants of northern Sweden [34]. A large number of students and trainees with various levels of clinical experience pass through the hospital.

The trauma teams consist of members from different departments. When a trauma case arrives at the hospital, team members leave their regular duties and assemble in the emergency room at the emergency department. In this study, the objective of the trauma team was to identify life-threatening injuries and initiate life-saving actions. The “patient” was a patient simulator, specifically a digitally controlled manikin, SimMan 3G (Laerdal, Stavanger, Norway), pre-programmed into a standardized case suffering from hypovolemia due to external trauma (injury severity score [ISS]: 25). The manikin was made up to simulate either a blunt abdominal trauma (i.e. a spleen injury) or a stab wound in the axilla. The observations of the team’s performance started after the patient was handed over by the ambulance personnel and the trauma team started their assessment. Time was an important constraint, as the team had to act to reduce the extent of the patient’s injuries and prevent secondary damage. The assessments of the patient simulator were based on current guidelines, and were aimed at systematic early identification of the injuries according to the Advanced Trauma Life Support (ATLS) system [35]. The team members had predetermined roles and positions in the emergency room according to the hospital’s standard operating procedures for trauma care.

### Data collection

The data collection took place in 2009–2010 and is described at greater length in earlier articles [36, 37]. Eighteen trauma teams were audio and video recorded at the emergency department during regular team training sessions. Three video cameras were placed in the emergency room to capture the team members’ positions and movements. Wireless microphones were used to individually register the verbal communications of each participant. The data were collected in F-Rex [38], a software program developed by the Swedish Defence Research Agency (FOI, Linkoping, Sweden), to reconstruct and investigate the incident. Field notes and observations were made by the first author (MHm). The team training was designed to last 15 min before the instructor interrupted the training.

### Data analysis

We used quantitative content analysis [39] to study how the team leaders used non-verbal communication. This method is often used to study communication in media. The advantage of using it in this case was that it allowed us to examine and categorize the large volumes of data it generated during the study. Instead of counting words, we counted time. In the first step, two of the authors (MHm and MJ) observed the video-taped trauma team training and identified three notable non-verbal positions that the leaders were using: gaze directions, speech time and gestures. A code scheme was constructed, and the two authors coded the first session separately. The results were thereafter discussed with the co-authors. Since there was a high degree of consistency in coding, we decided that one of the authors (MHm) should carry out the coding of the entire material. This method contains aspects of qualitative methodology, since the quantitative data are interpreted in relation to the context.

The team members’ positions and movements around the patient simulator in the emergency room were compared to the predetermined positions specified in the hospital’s standard operating procedures for trauma care, and any deviations from the predetermined positions during the training session were registered. Time sequences of the leaders’ communications, specifically gaze direction, speech time, and gestures, were identified to the level of seconds and then calculated as proportions (%) of the 15 min of training (cf. [40, 41]). Gaze direction was categorized into gaze directed towards the patient and the monitor, and gaze directed towards other team members and around in the emergency room (cf. [32]). Speech time was measured and calculated as proportion (%) of spoken time during the team training (cf. [42]). Vocal nuances during speech were categorized in terms of tone of voice (barely audible/whispering vs. shouting/high voice) and intonation. Gestures were categorized as to whether they occurred during silence (speech-independent conversational gestures) or during speech (conversational gestures).

## Results

The team leaders’ non-verbal communication is described below in terms of position and movement in the emergency room, gaze direction, vocal nuances, and gestures. Excerpts are inserted to illuminate the leaders’ communication and how they used vocal nuances to emphasize their message.

### Position around the patient in the emergency room

**Fig. 1:**
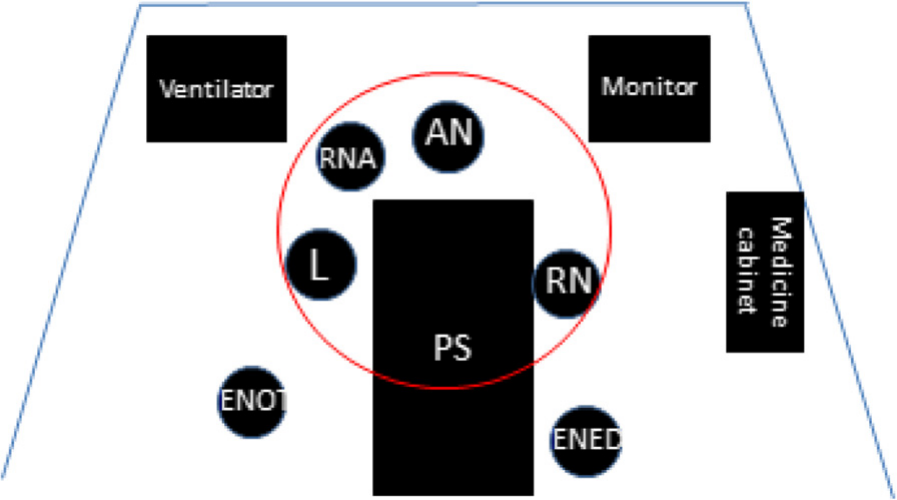
The “inner circle” around the patient simulator. AN = anaesthesiologist, ENED = enrolled nurse from the emergency department, ENOT = enrolled nurse from the operating theatre, L = leader, PS = patient simulator, RN = registered nurse, RNA = registered nurse anaesthetist

Only eight of the eighteen team leaders positioned themselves with an overview of the patient and the monitor with the patient’s vital signs (Fig. 1). Taking a position on the opposite side of the patient simulator hampered the leaders’ overview of the monitor, since this meant they had the monitor behind them. There was an invisible but clear division of the area around the patient simulator where the action took place, the “inner circle” (Fig. 1). Not all team members had obvious access to the limited space around the patient. The physicians’ positions were in the inner circle, but the nurses moved into and out of the area, and so the physicians had to step aside when the enrolled nurses needed to execute their tasks.

As noted above, since the area of the inner circle was limited, the team members had to step aside when other team members needed access to the patient to accomplish their tasks. These changes in position required both attention and collaboration, since the movements occurred without verbal requests. Generally, the team members seemed to have knowledge and awareness of each other’s tasks both in time (when) and space (where).

**Fig. 2:**
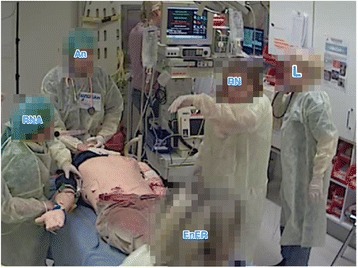
The leader is positioned behind the RN and hence blocked from assessing the patient simulator. AN = anaesthesiologist, ENED = enrolled nurse from the emergency department, L = leader, RN = registered nurse, RNA = registered nurse anaesthetist

**Fig. 3:**
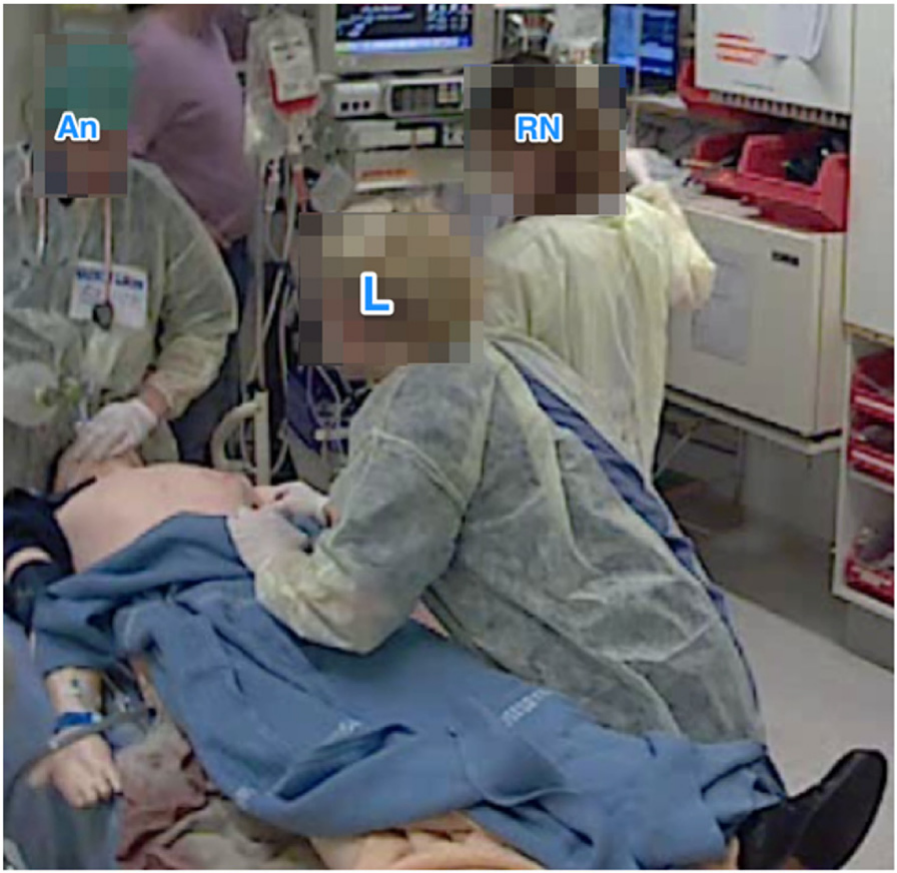
The leader has moved to the end of the bed; in this position, their assessment is impeded. AN = anaesthesiologist, L = leader, RN = registered nurse

In four teams (P, L, M, and R), the leaders positioned themselves outside the inner circle from the beginning of the team training. In three teams (E, M, and P), the leaders were blocked from the inner circle by other team members. In team E, the registered nurse from the emergency department positioned herself in front of the leader, and in order to gain access to the inner circle, the leader changed position from the right side of the patient simulator to the left. Subsequently this situation was repeated; this time, the registered nurse and the enrolled nurse from the emergency department interfered with the leader’s assessment and blocked the leader’s access to the inner circle. Now the leader changed position from the left side to the right side, but without being able to enter the inner circle. In Team P, the leader was prevented from reaching the patient simulator by the registered nurse. The leader tried to move around the registered nurse, but the registered nurse turned her back towards the leader and did not notice the leader’s effort to reach the patient. The leader was positioned behind the nurse (Fig. 2) or at the end of the bed, without access to the patient simulator in order to complete the assessment (Fig. 3).

### Gaze direction

The leaders directed their gaze towards the patient simulator and/or the monitor for more than 50 % of the time while assessing and examining the patient simulator (Table 1). In team A, the leader gazed towards the patient simulator and monitor 92 % of the time, while in teams C and E the leaders gazed in this direction about 58 % of the time. It was notable that the team members rarely made eye contact during the performance. Mutual gaze was established when the leader was discussing the priority of actions or treatment with the anaesthesiologist. Generally, the tasks were distributed by calling out, without directing the commands to specific team members (but rather to “someone”) and without establishing eye contact. A few leaders gazed around at the team, made eye contact, and pointed at team members when urgent tasks needed to be executed, such as using special equipment or calling the operating theatre. The leaders who gazed around at the other team members in the emergency room were also more silent.

**Table 1:** The proportion of time (%) during team training when the team leader’s gaze was directed toward the patient/monitor or around at the team members in the emergency room, when the leader was silent or speaking, and when the leader used gestures during silence or during speech

Team	Gaze directed towards patient or monitor %	Gaze directed around the ER or at the team members %	Silence %	Speech %	Gestures during silence %	Gestures during speech %
Team A	92	8	37	63	74	85
Team B	63	37	74	26	44	41
Team C	58	42	60	40	45	28
Team D	78	22	83	17	51	36
Team E	58	42	56	44	43	82
Team F	85	15	61	39	28	79
Team G	81	19	76	24	68	31
Team H	73	27	73	27	56	62
Team I	68	32	94	6	42	57
Team J	72	28	46	54	60	30
Team K	69	31	66	34	67	18
Team L	86	14	78	22	63	34
Team M	86	14	73	27	30	24
Team N	73	27	84	16	36	33
Team O	89	11	75	25	77	3
Team P	67	33	67	33	63	27
Team R	70	30	80	20	12	15
Team S	56	44	70	30	58	43

### Vocal nuances

The proportion of speech time during the team training varied between the team leaders. The leader in Team A spoke about 63 % of the time, while the leader in Team I spoke only about 6 % of the time (Table 1).

The leaders used vocal nuances together with gestures to underline and emphasize the importance of their commands. Excerpt 1 gives an example of this, as a leader summarizes the patient simulator’s injuries; bold text is used to show intonation and emphasis.

*Excerpt 1*

Enrolled nurse from ED: Shall we insert the catheter?

Leader: We’ll insert ca-/yes we should **not** insert the catheter, we can do that in theatre, this patient is still bleeding, **the most important thing** is to go to theatre and gain control of the bleeding (pause) but no bleeding out, we see that he has **a fast pulse**, he’s bleeding internally and we have no idea what it looks like (pause) an obvious case of laparotomy so **the most important thing** must be to go with to/with intravenous needles and fluids going and straight to theatre (pause) does anyone think we need to do something else before we go?

**Fig. 4:**
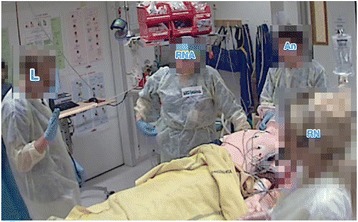
The leader uses a gesture (holding one hand up) to emphasize that the team should not insert the catheter. An = anaesthesiologist, L = leader, RN = registered nurse, RNA = registered nurse anaesthetist

In excerpt 1, besides emphasizing prioritization — “**the most important thing**” — the team leader got the team members’ attention by holding up one hand when making it clear that the team “should **not** insert the catheter” (Fig. 4). In pointing out vital signs that indicated life-threatening conditions, the leader emphasized the term “**fast** pulse”.

The leader in excerpt 2 used a loud and clear voice along with terms such as “**very fast**” and “**very important**” in order to reinforce and prioritize the team’s actions, and then continued to verbally emphasize the prioritization of the injuries and/or tasks. Bold text is again used to show intonation and emphasis.

*Excerpt 2*

Leader: Undressing is **very important** in this kind of for a patient (pause) so that we get it done quickly (pause) then you take samples (looking at the enrolled nurse from ED).

Leader: And then, ehh, start fluids early (pause) the goal with these patients must also be that we **very quickly** get to the logroll (pause) **very quickly** so that we have all the injuries clear to us (pause) both front and back of the patient **much earlier** than in a patient with blunt trauma (pause) so that’s the only thing that is different when we know there is a knife injury (pause) yes (pause).

The majority of the leaders with less access over the inner circle were generally more silent during the ongoing training (Table 1). Leaders who seemed to have less control over the inner circle were characterized by a soft-spoken or whispering voice, speaking in a hesitant voice, and giving hesitant answers.

### Gestures

**Fig. 5:**
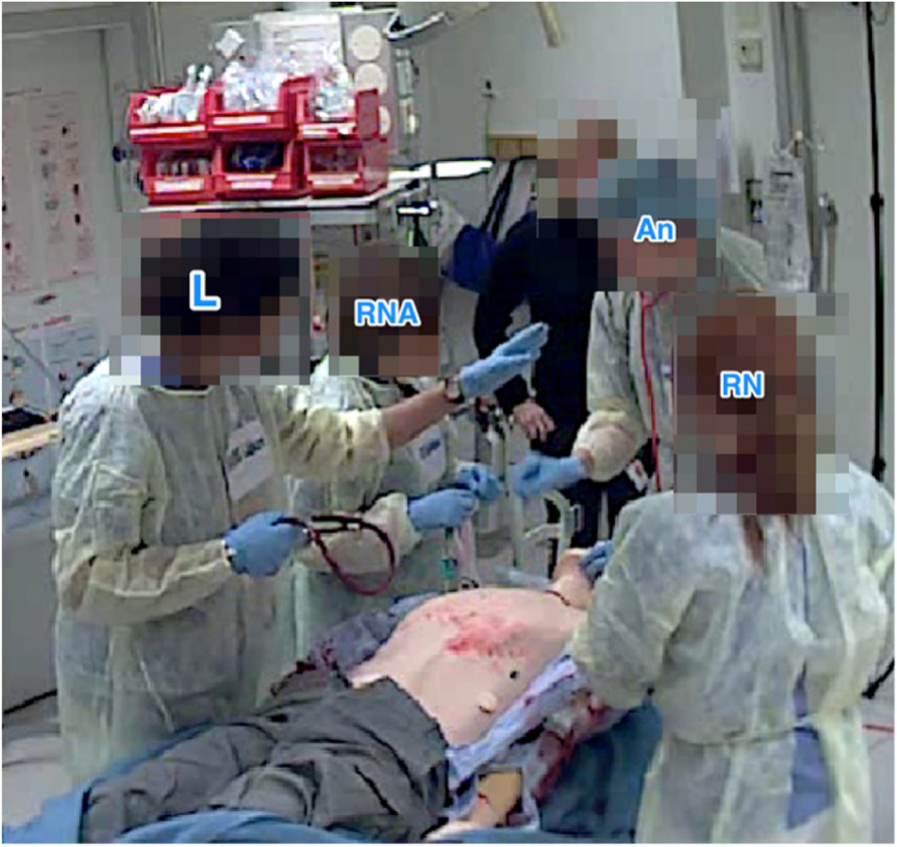
The leader uses gestures while distributing tasks, pointing at a team member to clarify the statement “You take responsibility for A [airway].” AN = anaesthesiologist, L = leader, RN = registered nurse, RNA = registered nurse anaesthetist

**Fig. 6:**
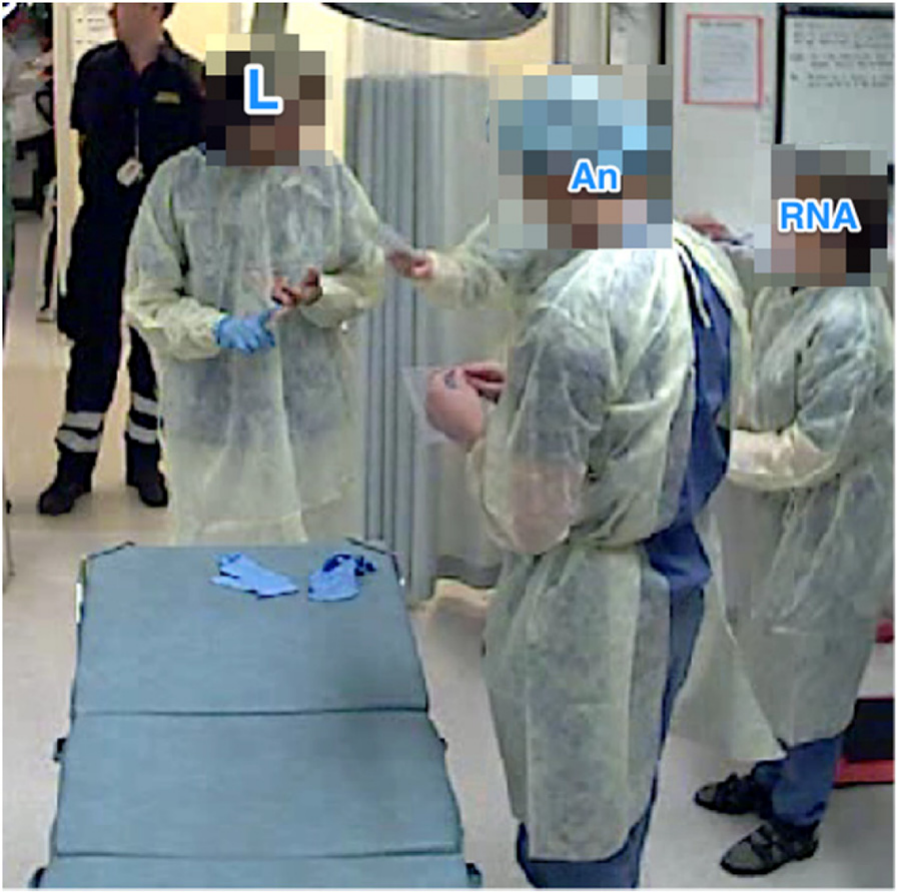
The leader summarizes the patient’s injuries to the team members, illustrating the number by counting on their fingers. AN = anaesthesiologist, L = leader, RNA = registered nurse anaesthetist

**Fig. 7:**
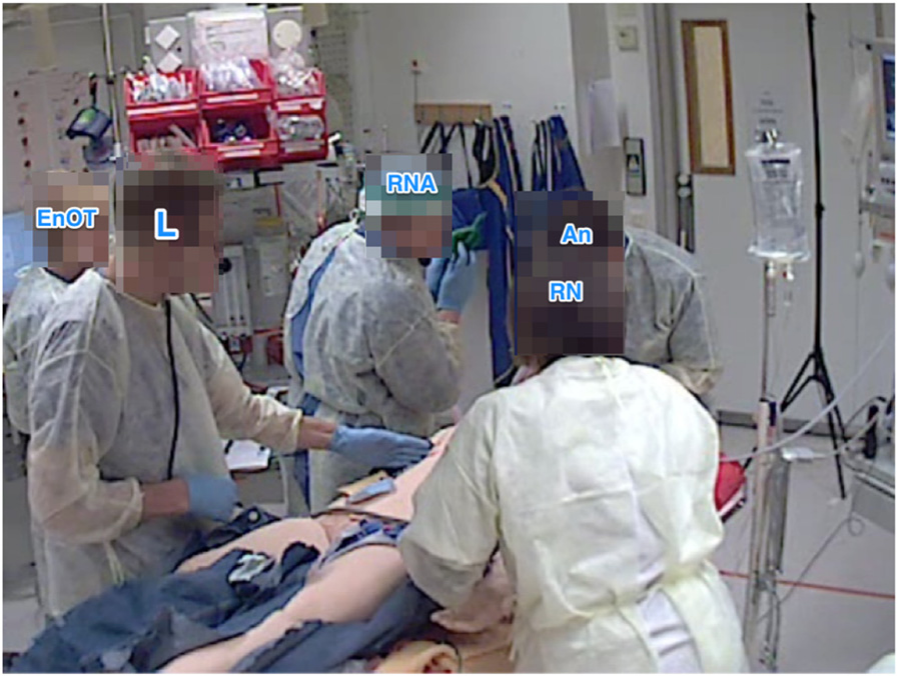
The leader summarizes the patient’s injuries, pointing towards the abdomen to emphasize the report. AN = anaesthesiologist, ENOT = enrolled nurse from the operating theatre, L = leader, RN = registered nurse, RNA = registered nurse anaesthetist

The leaders’ tasks were associated with hand movements of an exploratory nature when assessing and examining the patient simulator; for example checking the pulse, checking the temperature of the skin, and searching for fractures. The gestures seemed to be more important when urgent tasks were distributed or when the team members did not notice the leader’s task allocation. The leader used a hand (Fig. 5) or an index finger to point, and emphasized the command by saying “you”. The numbers of injuries were also clearly illustrated and summarized by the leader raising their hand and counting on their fingers (Fig. 6). Concise gestures were used to indicate where the injuries were situated on the patient simulator— the abdomen (Fig. 7) or the arm — and to show where the needle should be situated. The leaders who used more gestures when communicating also maintained their access to and control over the inner circle, even when they were standing outside it (Table 1). Generally, the team leaders remained silent during the assessment of the patient simulator. The leader in team A used their hands about 74 % of the time they were silent, while the leader in team F only used their hands about 28 % of their silent time (Table 1). In five teams (A, E, F, H, and I), the leaders gesticulated and emphasized their speech more than 50 % of the time.

Leaders who were silent, used few gestures, and gazed around at the team members during the team training had less access to and control over the inner circle. Distinct gestures that reinforced the leader’s message seemed to keep the team members’ attention and strengthen the leader’s position as the authority in the team.

## Discussion

The team leaders who gained control over the inner circle used clear non-verbal communication and positioned themselves as heads of the team. They solidified their verbal messages using gaze direction, vocal nuances, and gestures. Team leaders who positioned themselves (or were positioned) outside the inner circle were quiet, gazed around at the team members, and made little use of vocal nuances and gestures.

The inner circle is the most important space in the emergency room. In this space, the leader can see both the patient simulator and the monitor displaying vital signs. In teams where the leader had control over the inner circle, the members seemed to have an awareness of each other’s roles and tasks, knowing when in time and where in space these tasks needed to be executed. This has also been described in surgical teams, where coordination behaviours increased in critical situations [22, 23]. The coordination between the scrub nurse and the surgeon did not always involve verbal communication; rather, changes in the surgeon’s tone of voice and body shifts indicated a change in the urgency of the patient’s status [22]. Changes in the surgical intervention led to exchange of roles in the theatre. Moore et al [23] described how the senior surgeon changed position and took the intended role as the responsible surgeon when the registrar stretched and distanced himself away from the wound. According to the authors, understanding the nuances of non-verbal communication could not only improve teamwork but also increase patient safety, and team members who are aware of how they use their body to communicate can “fine tune” their performance [23]. The results from an observational study on anaesthesia crews showed that the crew members adapted their coordination behaviour depending on changing situational requirements. A shared understanding of task requirements could be assumed to be especially effective in high workload situations where verbal messages to coordinate actions may be limited [27]. Team members in anaesthetic teams have been described as conveying non-verbal cues that coordinated their activities while inserting the tracheal tube. Furthermore, the smooth teamwork was interrupted and difficulties became visible when inexperienced team members were involved in this procedure as the timing of synchronizing tasks was missing [21]. Our study also showed that leaders who retained access and control over the inner circle emphasized their commands with phrases that reinforced their message (e.g. “very important” and “much earlier”), used vocal nuances to underline the commands in loud and clear voices, and used gestures to clarify the prioritization of key tasks and to show where injuries were situated.

Deviations in the leaders’ communication increased the ambiguity in the communication, which had consequences for the teamwork. This became obvious for instance when the registered nurse blocked the team leader’s access to the inner circle and prevented the leader from continuing their assessments. Being positioned and/or taking a position outside the inner circle hampered the leader’s possibility to examine the patient and complete their tasks. It also meant that communication was diminished, since it required a stronger voice to give clear commands to enable the team members to perceive the message.

We also found that the team members conveyed non-verbal communication to coordinate the emergency activities in the team. Difficulties arose and become visible when more inexperienced team members were involved in the coordination of the activities. This emphasizes the importance of taking into consideration the fact that high-performing teams need to be flexible depending on the patient’s condition, which can abruptly deteriorate into a critical situation. In addition, the leader’s actions are dependent on the team members’ knowledge, experience, and ability to think ahead [22, 23]. Even if the leader is aware of the patient’s condition, there may be team members who do not have the knowledge or experience to perceive the critical situation. It becomes important for the leader to create a shared perception of the situation and how to solve the problem. In “ideal” trauma team work, all members will know what they must do, but the leader cannot take this for granted [9].

In the present study, a majority of the leaders remained silent for more than 50 % of the ongoing trauma team training, and in one team the leader was silent for 94 % of the scheduled time. It is possible that these silent leaders were more uncertain about their own knowledge, and were expressing this in their communication. Silence should not only be regarded as the absence of communication and the opposite of speech [43]. There might be multiple and complex relations, also connected to power relations, that may constrain the communication within teams [44–46]. Silence can also be an avoidant conflict style, associated with attempts to protect one’s self-image within a strong hierarchical structure without being exposed as lacking in knowledge [47, 48]. The hierarchical structure in the medical discourse can contribute to participants’ silence (and/or avoidance) rather than a participatory teamwork communication [49].

In another study based on the same material [37], we found that the team leaders’ verbal communication strategies were flexible and that the leaders changed positions depending on the severity of the patient’s condition and the interaction between team members. When the leaders communicated knowledge and explained their priorities, they positioned themselves as authoritarian leaders; and when they discussed the situation with the team members, they were positioned as more egalitarian leaders. In urgent situations, directed commands were required, and the leader’s position as authority become obvious. In the present study, we found that the leaders’ non-verbal communication was also flexible in their interactions during the trauma team training. The leaders varied their non-verbal communication due to the urgency of the situation. When they called out commands, they also reinforced these commands with gestures, and sought eye contact.

In emergency situations, the trauma team leader ensures that work is performed in accordance with existing routines, meaning that the leader should organize the teamwork effectively to ensure early treatment. This study puts not only verbal communication, but also non-verbal communication, in focus in the interdisciplinary trauma team. We have shown that non-verbal communication is important for the leader’s performance. It appears that some leaders occupy the obvious position in the team by having a clear communication, both verbal and non-verbal. Other leaders conveyed a degree of uncertainty, and emphasized the ambiguity of their verbal communication with a hesitant, vague, or non-existent non-verbal communication. Personality and experience are important for how leaders act, but awareness of how to communicate, training, and practice are essential components for developing an effective authoritarian leadership as a trauma team leader. Understanding the nuances of non-verbal communication could improve teamwork. Team members who are aware of how they use their bodies to communicate will be able to facilitate and improve their performance. This is even more important for inexperienced leaders, since ambiguous verbal communication amplifies the ambiguity of non-verbal communication, which can lead to compromising the safety of the patient.

This study has identified deficiencies in leaders’ non-verbal communication that lead to ambiguity in the teamwork and probably delay the care of the severely injured patient. Communication cannot be taken for granted; it needs to be practiced regularly just as technical skills need to be trained. Simulation training provides healthcare professionals as well as medical and nursing students the opportunity to put both verbal and non-verbal communication in focus, in order to improve patient safety. It is therefore important to further study trauma team communication, preferably during real emergency events. There is a lack of studies on how characteristics such as profession, gender, and ethnicity affect the communication (both verbal and non-verbal) in interdisciplinary teams. More studies are needed, preferably performed during real emergency cases, to verify our results.

### Limitations of the study

Some limitations of the study should be discussed. First, the study setting was not a real emergency event. However, the simulated situation was based on a real event, and efforts were made to create as realistic environment as possible. The trauma team training was conducted in an authentic environment at the emergency department, with the participants acting in their own roles as part of the trauma team, and the patient simulator was treated according to the hospital’s standard procedures and ATLS guidelines. There are also both strengths and weaknesses in the analysis of the video recording. The major advantage is that the recordings allowed us to repeatedly observe the audio-and video-recorded material. However, the cameras did not always include the entire field of view in the emergency room, and could not record events occurring off the screen [49]. The participants’ work was performed within a limited area around the patient simulator, meaning that other team members could stand in the way of the camera and thereby complicate the analysis. In this study, we used three different cameras in order to avoid this obstacle and to capture all participants in the team. Another important issue discussed within the research team was the question of whether ethnic and cultural differences could have been of importance. The material in this study was too limited to draw any conclusions regarding this matter, but future studies focusing on ethnic and cultural differences between team leaders would be of importance and interest.

### Implications

There are several potential benefits to be gained from an increased understanding of the role of non-verbal communication in the trauma team. Team members who are aware of their non-verbal communication can improve their performance. In education, trauma team training, and for all professionals working in trauma teams, it is important to take these circumstances into account since they can ultimately affect the care of the patient.

## Conclusions

The main conclusion in this study is that non-verbal communication reinforced the team-leaders’ communication. Team leaders with access to the inner circle used gestures to reinforce and emphasize their verbal communication, in contrast to the leaders who were silent, and did not use gestures, and were positioned and/or positioned themselves outside the inner circle. There is already a great awareness of the importance of verbal communication in trauma teams. This study deepens our understanding of the importance of non-verbal communication, and shows that non-verbal communication should also be taken into consideration in the education of trauma team leaders. The non-verbal communication also reinforced the team leaders’ deficient verbal communication.
